# Effective community mobilization: Evidence from Mali^☆^

**DOI:** 10.1016/j.worlddev.2025.106956

**Published:** 2025-06

**Authors:** Maria Laura Alzua, Juan Camilo Cardenas, Habiba Djebbari

**Affiliations:** a Universidad Nacional de la Plata - Conicet, CEDLAS and PEP, Argentina; b Universidad de los Andes, Chile; c Aix-Marseille Univ., CNRS, EHESS, Central Marseille, IRD, AMSE, Marseille, France

**Keywords:** Public good provision, Behavioral experiments, Community-based development, Sanitation

## Abstract

Experts argue that the adoption of healthy sanitation practices, such as hand washing and latrine use, requires focusing on the entire community rather than individual behaviors. According to this view, one limiting factor in ending open defecation lies in the capacity of the community to collectively act toward this goal. Each member of a community bears the private cost of contributing by washing hands and using latrines, but the benefits through better health outcomes depend on whether other community members also opt out of open defecation. We rely on a community-based intervention carried out in Mali as an illustrative example (Community-Led Total Sanitation or CLTS). Using a series of experiments conducted in 121 villages and designed to measure the willingness of community members to contribute to a local public good, we investigate the process of participation in a collective action problem setting. Our focus is on two types of activities: (1) gathering of community members to encourage public discussion of the collective action problem, and (2) facilitation by a community champion of the adoption of individual actions to attain the socially preferred outcome. In games, communication helps raise public good provision, and both open discussion and facilitated ones have the same impact. When a community member facilitates a discussion after an open discussion session, public good contributions increase, but there are no gains from opening up the discussion after a facilitated session. Community members who choose to contribute in the no-communication treatment are not better facilitators than those who choose not to contribute.

## Highlights

• The adoption of healthy sanitation practices in rural areas requires focusing on the entire community’s behavior.
• One limiting factor in ending open defecation lies in the capacity of the community to collectively act toward this goal.
• The benefits of sanitation through better health outcomes depend on whether other community members opt out of open defecation.
• A series of experiments are designed to measure the willingness of community members to contribute to a local public good.

## 1 Introduction

Overall disappointment with top-down policy led to the inclusion of participatory approaches in development programs (Chambers, 1983). Community-based programs rely for the most part on community mobilization to encourage behavioral change (parent teaching associations, village health committees, community-based targeting of social programs, etc.). Possible mechanisms for success include social learning, emergence of shared norms and exploitation of local complementarities. A successful community mobilization can generate support for a local development issue and a reordering of priorities that result in immediate actions. Failure may occur because the public arena is captured by interest groups opposed to change or because of a lack of capacity to plan actions to implement the desired change. The existing evidence on the effectiveness of community-based development projects is rather mixed (Mansuri and Rao, 2004, Mansuri and Rao, 2012).

Community-Led Total Sanitation (CLTS) is a type of community-based intervention that aims to eliminate open defecation (OD) by engaging local communities to build latrines and use them. CLTS became a flagship program of the international community to support improved sanitation in rural areas in several developing countries in Sub-Saharan Africa and South Asia. However, the success of CLTS varied considerably (Wolf et al., 2022, Cameron et al., 2022) according to physical and sanitary factors (population density, geography, or initial burden of the disease). It is likely to be shaped by social factors, such as the initial local capacity for collective action, but also by the actual power of the participation process to induce collective action. Lack of mobilization may result in low adherence to CLTS and adversely affect its effectiveness. However, little is known about the process through which communities are mobilized, which is central to CLTS, as well as other community-based interventions.

In this paper, we seek to open the “black box” of the participatory approach in CLTS using a laboratory-in-the-field experiment designed to reproduce some of the features of the community mobilization process of the Mali CLTS programme.^1^ CLTS Mali relies heavily on community mobilization to trigger behavioral change toward safer sanitation practices (Kar and Chambers, 2008).^2^ Our focus is on two particular processes. First, participation in the provision of safe sanitation is initiated through the convening of a community meeting during which an external facilitator introduces the subject (sanitation) and allows a public discussion to take place. One purpose of the initial round of discussion is for the external facilitator to identify a community champion, someone who clearly understands the socially desirable goal (ending open defecation) and the individual actions needed to achieve it (using toilets equipped with a slab and a hand washing device). In the second stage, the external facilitator delegates to the community champion the authority to lead the community discussions, relying on this person to emphasize the collective goal and explain how to reach it.

We are interested in evaluating the potential of the two communication strategies to enhance collective action. We expect communication to help coordinate individual decisions towards the provision of safe sanitation. In evaluating the design options of the participatory method, we pose the following inquiries: What is the magnitude of the collective gains achieved when engaging the community in discussions about the commons problem? Secondly, are discussions facilitated by a community member more effective than the non-directed open discussions? Thirdly, does a community member who demonstrates a willingness to support collective action make an effective leader, *i.e.*, someone who can trigger a higher willingness to support it in the community? Lastly, how important is the selection of a sanitation champion for the effectiveness of directed discussions?

We offer quantitative evidence on these questions using tools from experimental economics. We designed and conducted a series of experiments in the field in 121 villages representative of rural Koulikoro in Mali, a province the size of Portugal. Each series brought together an average of 18.5 community members. We devised a simplified public good contribution game for which the decision to make is to contribute or not to a local public good. We measured individual willingness to contribute in a series of three treatment conditions. During the first round, community members make their contributions without communicating. In the next two rounds, two communication treatments were introduced: undirected open-group discussion and directed-group discussion. The discussions in these two rounds lasted for the same time before the participants privately made their decisions (decisions were kept confidential until the end of the series of experiments). To conduct the directed group discussion, the experimenter randomly selected a member of the community (game participant). This member was instructed about the optimal group solution and asked by the experimenter to share the information with the rest of her group.

Our games differ from CLTS in many ways. We highlight here two main ones. First, decisions and rewards are private in the games; sanitation facilities and behavior are partially observed by others. Hence, the actual impact of CLTS on sanitation may reflect the social pressure that those who equipped their homes with private latrines exerted on those who did not (*e.g.*, by pressuring them to adopt the collectively beneficial action). We do not capture this important mechanism with our game design.^3^

Second, in practice, CLTS community champions are not randomly selected by the external facilitator; they self-select into this role. In the games, they are randomly selected. Selecting them at random allows us to test whether those who were intrinsically motivated and contributed in the absence of any discussion triggered the same gains as those who did not. However, this choice of design can potentially limit the external validity of the study findings. An issue in interpreting the evidence is that the incentives faced by the person randomly selected to direct discussions in the local public good setting are different from those faced by the selected leader in the actual CLTS. In the former, this person always has an incentive to try to convince others to contribute. In CLTS, some community members may actually wish to dissuade others from following the message advocated by CLTS. In this case, initially engaging the community in an open dialogue may, in fact, help identifying a sanitation champion to lead community discussions. If there is broad agreement in the community on the importance of improved sanitation, testing this hypothesis provides insights into the relevance of initial open discussions and the choice of a community leader motivated to support the public good.

The existing literature evaluating participatory approaches to development in general Casey (2018), and community-based sanitation programs in particular Venkataramanan et al. (2018), offer various explanations for success or failure but limited evidence on the underlying mechanisms. Although there is a small thread of literature that contrasts participatory to top-down approaches (Alatas et al., 2012, Alatas et al., 2014, Madajewicz et al., 2021, Olken, 2007), there is growing interest in assessing interventions that aim to induce more or better informed participation (*e.g.* Bjorkman and Svensson, 2009, Allakulov et al., 2023, Arkedis et al., 2021, BenYishay et al., 2022). Our research contributes to the latter by examining how key features of a well-established community-based sanitation programme improves participation. In doing so, we take the social planner’s objective (ending OD) as given and assess the actual policy design choices made using a behavioral public policy evaluation approach (Jacquemet and L’Haridon, 2018).

This study has the potential to offer several insights into the design of a prototypical participatory approach aimed at increasing the provision of local public goods. Community discussions mobilize individuals by increasing their willingness to contribute to end OD. If only one method of communication were to be used, the relative gains from open group discussion and directed discussions would be similar. Directed discussions on their own are found to be as effective as the combination of directed and undirected discussions, whereas undirected discussions alone is not as effective as the combination. This suggests that making clear the actions needed to achieve the socially desirable goal is crucial to achieving the collective gains. Moreover, a policy that values community members’ time should prefer a strategy based on directed discussions to the one based on open then directed group discussions. These insights hold even when community members leading directed discussions are randomly selected, provided that everyone agrees that improved sanitation is welfare-improving. In this case, a community member who shows higher willingness to support local public goods provision is not more effective in increasing the contributions of others than one who does not. In a setting where there is disagreement on whether ending OD is desirable, holding an open discussion can be useful to select a sanitation champion and could lead to even higher gains from directed discussions.

The remainder of the paper is organized as follows. Section 2 describes the CLTS-Mali participatory approach and draws predictions on how participation may affect local public good provision. In Section 3, we present the data and methods. We report the findings in Section 4. Section 5 offers a summary and discussion of the findings and Section 6 concludes.

## 2 Illustrative example and predictions

In this section, we provide the descriptive elements in the CLTS approach that motivate the design of our experiment and the hypotheses that will be tested in the empirical section.

### 2.1 A prototypical participatory approach to local public good provision

Experts argue that the adoption of good sanitation practices requires focusing on the entire community rather than individual behaviors (Kar and Chambers, 2008).^4^ According to this view, the limiting factor in ending open defecation is neither informational nor technical or financial, but lies in the capacity of the community for collective action. CLTS aims to enhance this capacity by drawing the attention of community members to the local sanitation situation, providing them with the space to discuss it and to decide whether they individually want to contribute to ending OD.

Throughout this paper, we rely on CLTS as carried out in Mali as an illustrative example. In previous research, using a village-level clustered randomized controlled trial design, we found massive improvements in household sanitation, as well as evidence of improved child growth (Pickering et al., 2015). In Alzúa et al. (2020), we found that information about the health benefits of safe sanitation did not play a role in the success of the intervention. We found no difference in knowledge about the risks associated with poor sanitation in intervention and non-intervention villages. In both groups, households were already quite knowledgeable about these risks. Cameron et al. (2022) combines data from different locations (India, Indonesia, Mali, and Tanzania) to analyze the effects of CLTS variants. All but the Mali experiment show modest impacts.

Unmanaged human waste spreads diseases (non-rivalrous and non-excludable). The objective of CLTS is to end open defecation one community at a time. To achieve this objective, CLTS works to stir up collective action so that every household in the community is equipped with and starts using a basic private latrine. The main activity of CLTS consists of a 3-hour community mobilization facilitated by external CLTS-trained agents. CLTS agents are trained not to teach / educate or tell people what to do, but rather “facilitate communities to take a decisive role in ensuring that each and every member internalizes the implication of poor sanitation.” (Handbook on CLTS, Kar and Chambers, 2008). By involving a community champion, the CLTS participatory approach attempts to mimic as much as possible a spontaneous endogenous process of participation with minimal inducement by an external facilitator.

During this initial community gathering, several activities are carried out to assess the risks associated with open defecation. The activities are meant to bring attention to sanitation (*e.g.*, mapping of open defecation places, showing of contamination of food through flies, etc.) and to make salient the costs of unhealthy sanitation and hygiene practices. This is the first stage of the community mobilization process, providing space for open and public discussions on the sanitation situation.

These activities carried out around sanitation trigger discussions between community members, making it possible for CLTS facilitators to identify sanitation champions among community members. Facilitators then prompt these community champions to address the community, showing their commitment to build a latrine, and urging others to follow this course for the sake of the whole community. We model this stage as a stage of directed community discussions, led by the community member identified as a sanitation champion. CLTS staff select a person who expressed a willingness to contribute to end OD to lead the directed discussions. Other factors such as charisma and leadership skills may also be observable to them. Finally, at the end of this address, community members are encouraged to volunteer to publicly commit to building, repairing, and using latrines, with written details on the plan of action; see Alzúa et al. (2020) for details on CLTS Mali.

CLTS is a relevant illustrative example of a participatory intervention aiming at solving a classic example of a collective action problem, whereas each member of the community bears the private cost of contributing by building and using latrines, and the benefits through better health outcomes depend on what the rest of the group does. CLTS narrative is that open public community discussions, as well as those led by community champions, are key drivers of behavioral change during the community mobilization process.

Community discussions may be providing a way for people to coordinate actions towards the socially beneficial outcome. When people have heterogeneous thresholds for conditional cooperation (Fischbacher et al., 2001, Chaudhuri, 2011), their willingness to contribute to a public good depends on the fraction of the group that does, with a threshold that differs between individuals. Engaging in discussions could serve as a means of coordination, potentially leading to higher contributions. If these discussions are guided by a community member who has a clear message to deliver, this person could potentially enhance contributions further.

### 2.2 Hypotheses

Free riding in collective action problems is not as prevalent as standard economic theory predicts (see, *e.g.*, Ledyard, 1995, Zelmer, 2003; and Chaudhuri, 2011, for reviews). Existing evidence from experimental economics shows that contributions to a local public good increase with communication (see, *e.g.*, Isaac and Walker, 1988, Sally, 1995, Cason and Khan, 1999, Bochet and Putterman, 2009). In CLTS, discussions are described as a key element for community mobilization to end OD. Hypothesis 1 is set to test if discussions are a mechanism resulting in a higher willingness to contribute to a local public good.

Hypothesis 1 Communication leads to higher contributions to the local public good.

According to the existing experimental literature, as participants become more experienced in repeated rounds, their contributions to local public goods decline. In CLTS Mali, community members are first exposed to open discussions and then to directed ones. The decline in contributions associated with learning effects may be offset by the increase associated with communication.

Hypothesis 2 Learning effects do not depend on whether the group is first exposed to directed or undirected discussions.

With Hypothesis 2, we are only interested in whether the order of the exposure to these two treatment conditions affects the overall gains from the two communication methods, *i.e.* we test for the absence of differential learning effects. Although the CLTS approach follows a two-stage communication strategy, we also test if the effect of directed discussion is the same as the effect of undirected discussions (Hypothesis 3). The evidence should help in selecting the most effective communication method if only one of them was to be implemented.

Hypothesis 3 Directed discussions are as effective as undirected ones.

We also assess the gain from adding a directed discussion after the open discussion, or from adding an undirected discussion after a directed one. Rejecting Hypothesis 4 would require us to reconsider the rationale for having a two-stage discussion process.

Hypothesis 4 For each communication strategy, there is no value-added from combining it with the other strategy.

Finally, we are interested in distinguishing between two explanations for the gain in contributions resulting from directed discussions. Community champions may be good at convincing others to contribute (a selection effect); they may be effective because the experimenter instructed them to focus the discussions on the socially optimal decisions.

Hypothesis 5 The gains from directed discussions depend on the intrinsic willingness to contribute to the local public good of the community members chosen to lead the directed discussion.

## 3 Data and methods

### 3.1 Study participants

**Fig. 1:**
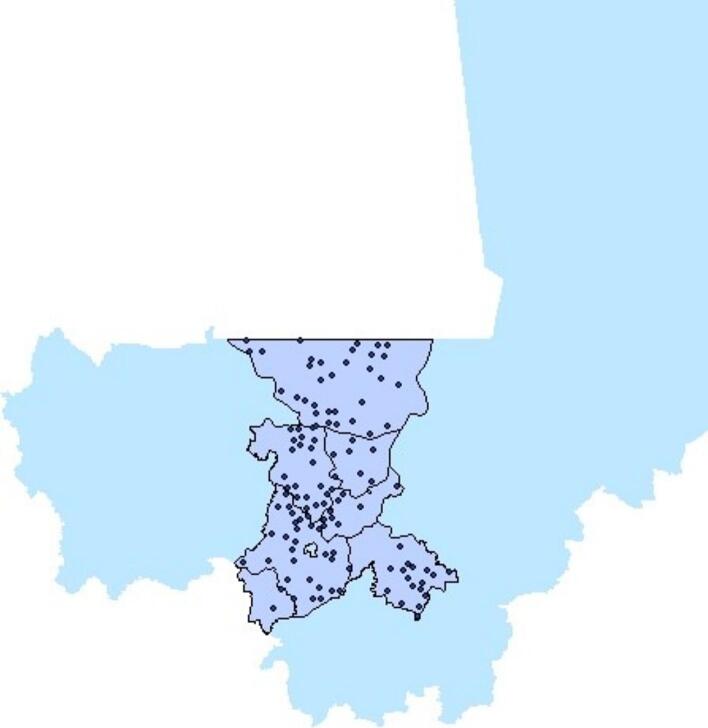
Study villages in the province of Koulikoro, Mali.

Participants in the experimental games are drawn from study villages sampled to assess the impact of CLTS in Mali. The sample includes 121 small rural villages in the Koulikoro region with low latrine coverage and no sanitation program in place. Fig. 1 shows the map of Mali with the province of Koulikoro highlighted. The dots indicate the location of the study villages. A timeline for the intervention and research activities is provided in the Appendix (Table A.1).

Based on the endline survey, 18 months after the intervention, 90 % of the households recalled that a member attended the community mobilization meeting (Table 1). Almost 90 % of them could remember the contamination experiment (showing how flies move from feces to food and water), closely followed by public commitments to build private latrines, the mapping of the village and OD places.

**Table 1:** Participation in and recall of CLTS activities.

	Mean (SD)
Household member present at CLTS meeting	0.90
	(0.30)
Man present at CLTS meeting	0.79
	(0.41)
Woman present at CLTS meeting	0.86
	(0.34)
Recall mapping village	0.83
	(0.38)
Recall mapping OD places	0.83
	(0.38)
Recall estimation of quantity of feces	0.70
	(0.46)
Recall show of contamination pathways	0.89
	(0.32)
Recall tour of shame	0.79
	(0.40)
Recall public commitments	0.84
	(0.36)
Recall videotaping	0.81
	(0.39)
Observations	7529

We conducted 363 sessions (3 per village) with 2,237 players. Each session gathered.

18.5 players on average. In each of the villages, after completion of the endline survey (April-June 2013), each of the respondents played a lottery with at least a 50 % chance to receive an invitation to a series of three experimental sessions.^5^ Attendance is voluntary and invited households choose an adult family member to represent them. There are 4,101 eligible households with at least one adult member (age 15 and older), of which 2,237 sent a household member to participate in the experiment.

Although households are randomly selected to participate in the games, the selection within the household is not random. This actually reproduces the way most community- driven programs operate: households get invited to participate in community meetings, and it is the household who chooses who to send. In Table 2, we compare the average characteristics of the game participants and adult non-participants of the same household, as well as the adult members of non-participating households. Invited households are more likely to send a woman, spouse of the head, married who usually works but did not in the past week. The higher female participation in the experimental sessions is consistent with the higher participation in the CLTS meeting activities in the intervention villages (Table 1). Illiteracy raised a practical challenge in designing the experiment; see the next section for how it was handled. The gap between individual participants and non-participating members of the same household is similar to the gap between individual participant and nonparticipating members from nonparticipating households.Table 3 and Table 4.

**Table 2:** Descriptive statistics on game participants and non-participants.

	(1)	(2)	(3)
Speaks bambara	0.73	0.75	0.77
	(0.44)	(0.44)	(0.42)
Head of household	0.24	0.30	0.29
	(0.43)	(0.46)	(0.46)
Spouse of the head	0.69	0.24	0.37
	(0.46)	(0.43)	(0.48)
Other household member	0.07	0.46	0.34
	(0.26)	(0.50)	(0.47)
Sex: male	0.25	0.55	0.47
	(0.43)	(0.50)	(0.50)
Religion: muslim	0.86	0.86	0.90
	(0.34)	(0.34)	(0.30)
Married	0.93	0.60	0.71
	(0.25)	(0.49)	(0.46)
Literate	0.14	0.14	0.14
	(0.34)	(0.35)	(0.34)
Occupation: farming	0.24	0.33	0.31
	(0.43)	(0.47)	(0.46)
Occupation: herding	0.02	0.04	0.04
	(0.13)	(0.19)	(0.19)
Occupation: merchant	0.03	0.04	0.04
	(0.18)	(0.18)	(0.19)
Occupation: construction	0.07	0.14	0.12
	(0.26)	(0.35)	(0.33)
Occupation: forestry	0.06	0.05	0.04
	(0.23)	(0.21)	(0.20)
Wage labor	0.01	0.02	0.01
	(0.12)	(0.14)	(0.11)
Family labor	0.62	0.59	0.59
	(0.49)	(0.49)	(0.49)
Self-employed	0.37	0.39	0.40
	(0.48)	(0.49)	(0.49)
Number of hours of work in the last week	37.06	34.98	35.12
	(16.27)	(16.27)	(15.35)
Usually work but not in the last week	0.25	0.07	0.08
	(0.43)	(0.25)	(0.27)
No. of organization	2.08	2.20	2.09
	(1.20)	(1.22)	(1.23)
No. of position of power	0.76	0.86	0.74
	(1.02)	(1.06)	(1.03)
Household assets index	0.44	0.46	0.45
	(0.15)	(0.15)	(0.15)
Observations	2,239	5,604	6,349

Notes: Mean and SD in parenthesis. (1) Individual participant. (2) Non-participant member in participating household. (3) Non-participant individual in non-participating household.

**Table 3:** Comparison: random leaders vs. other game participants.

	Random leaders	Other participants
Speaks bambara	0.73	0.76
	(0.44)	(0.43)
Head of household	0.24	0.21
	(0.43)	(0.41)
Spouse of the head	0.69	0.70
	(0.46)	(0.46)
Other household member	0.07	0.08
	(0.26)	(0.28)
Sex: male	0.25	0.24
	(0.43)	(0.43)
Religion: muslim	0.86	0.89
	(0.35)	(0.32)
1: Married	0.93	0.92
	(0.25)	(0.28)
1: Literate	0.13	0.17
	(0.34)	(0.38)
Household assets index	0.44	0.45
	(0.15)	(0.15)
Farm assets index	0.57	0.58
	(0.20)	(0.21)
Livestock index	21.53	25.12
	(33.42)	(42.77)
Index of locus of control	−0.28	−0.27
	(0.60)	(0.65)
Index of social capital	2.89	2.84
	(0.71)	(0.74)
No. of organization to which household belongs	2.08	2.15
	(1.19)	(1.38)
No. of positions of power held by household	0.76	0.81
	(1.02)	(0.99)
1: if anticipates everyone else to contribute (round 1)	0.49	0.43
	(0.50)	(0.50)
1: if contributes to foroba (round 1)	0.72	0.71
	(0.45)	(0.45)
Observations	98	2,139

Notes: Mean and SD in parenthesis.

**Table 4:** Testing randomness of leader.

	1: if leader; 0 if not
Speaks bambara	0.005
	(0.028)
Head of household	−0.016
	(0.025)
Spouse of the head	0.011
	(0.026)
Sex: male	0.016
	(0.021)
Age	0.000
	(0.000)
Religion: muslim	0.016
	(0.015)
1: Married	−0.017
	(0.024)
Literate	0.013
	(0.014)
Household assets index	0.015
	(0.041)
Farm assets index	−0.009
	(0.031)
Livestock index	0.000
	(0.000)
Locus of control index	0.001
	(0.009)
Social capital index	−0.009
	(0.007)
No. of organization to which household belongs	0.004
	(0.006)
No. of position of power held by household	−0.002
	(0.005)
1: if anticipates everyone else to contribute (round 1)	−0.012
	(0.010)
1: if contributes to foroba (round 1)	−0.001
	(0.011)
Constant	0.039
	(0.043)
Observations	2230

Notes: Mean and SD in parenthesis.

In the following, we first present the design and implementation of our experiment. We aim to reproduce selected features of the community mobilization process. Our main outcomes of interest are experimental contributions to a local public good. Our experimental subjects are individual members of the community.

### 3.2 Experimental design

The game under no-communication.

We designed a discrete public-good game following Marwell and Ames (1979). The choice of a discrete version of the public good game is motivated by the challenges regarding literacy and for the sake of simplicity (Cardenas and Jaramillo, 2007). Participation does not require the use of a pencil. There are two goods, a private and a public one, and *m* participants. Each participant was invited to participate in this game under three treatment conditions. The base treatment condition is a standard public good game without communication. In the other two treatments, we allow communication in the form of cheap talk. In the undirected group discussion, participants are allowed to talk to each other freely for five minutes. In the directed group discussion condition, a randomly selected participant is designated as the facilitator. The experimenter tells them what actions everyone should take in order to maximize the group payoff, and they are instructed to convey this message to the rest of the group.

The experimenter provides each participant $i=\left\{1,\cdots ,m\right\}\;$ with one token. The choice set includes two actions $x_{i}=\left\{0,\;1\right\}$, keeping the token ($x_{i}=0$) or investing it in the public good ($x_{i}=1$). If the token is kept, it yields a payoff p to player *i* only. If the token is invested in the group project, it yields a payoff of a to each player *j*, including *i.* The payoff function is given by: $y_{i}=p\left(1-x_{i}\right)+a\left(\sum_{j=1}^{m}x_{j}\right)$. Thus, the public good produced depends linearly on each individual decision to contribute. All decisions are made simultaneously and privately, without knowing what others will do. Assuming that participants only care about their monetary rewards and as long as a *<* p, there will be no incentives to contribute to the group account, *i.e.*, $\forall i=\left\{1,\cdots ,\;m\right\}\;:x_{i}^{Nash}=0$, resulting in a socially inefficient outcome. In this case, each player obtains $y_{i}=\;p$, and the group outcome is $\sum_{i=1}^{m}y_{i}=mp$. However, if every player contributes to the group account, *i.e.*, $\forall i=\left\{1,\cdots ,\;m\right\}\;:x_{i}^{soc.opt.}=1$, then the social optimum is obtained. In this case, the earnings for each player are $y_{i}=ma$, and the group outcome is $\sum_{i=1}^{m}y_{i}=m^{2}a>mp$. In our experiment, we set a=1 and p=10. At least 11 participants participated in the experiment in each village (an average of 18.5 participants per village). We refer to this game as the base game.

The communication conditions.

The communication rounds follow a first round of the public good game during which participants are not allowed to communicate with each other before making their individual contribution decisions. Each participant is then exposed to two variants of the base game for which communication is allowed. In the **undirected communication** condition, participants can have a 5-minute discussion before privately making their individual contribution decision. In the **directed communication** condition, a “community champion” is randomly chosen among the participants. This person is instructed to moderate a 5-minutes discussion and explicitly told that when every participant contributes the group maximizes its gains.^6^ After the 5-minute address by the “community champion”, participants privately make their contribution decisions.

Because we expected that the order in which groups of participants were exposed to the two communication conditions may matter for contributions, we randomly manipulate their order across groups of participants (randomization at the village level). This design choice allowed us to test whether the sequence in which groups were exposed to the communication conditions affected overall gains from the two communication conditions (Charness et al., 2012). Individual contributions are kept private and confidential until the end of the three rounds. At that time, the experimenter announces the total amount of contributions for each round and payments are made privately to each participant.

### 3.3 Framing and implementation of the experiments

There was a wide range of possibilities to present the games to the participants, from an abstract game without reference to a particular cooperation problem to a heavily framed situation that can hint the participants to the bigger purpose of the study, *i.e.*, studying their behavior to understand how the community-driven sanitation program may have affected them. Here, we chose a weak framework that serves to have participants’ minds set on collective action. This mild framing is intended for villagers to act according to past experience and underlying social norms.

Our public good games are framed as *foroba* games (*i.e.*, common pot in the local language). The name given to the token is *niyoro*, also a Bambara term for a token used in common transactions. Use of *foroba* and *niyoro* as labels should remind them of a familiar setting in which people usually contribute to a common pot and get a valuable amount in return. We decided against framing the public good according to the sanitation issue that is central to the research project in order to not contaminate our results with specific issues with the intervention that took place. However, we maintain a weak framing to collective action.

**Fig. A1:**
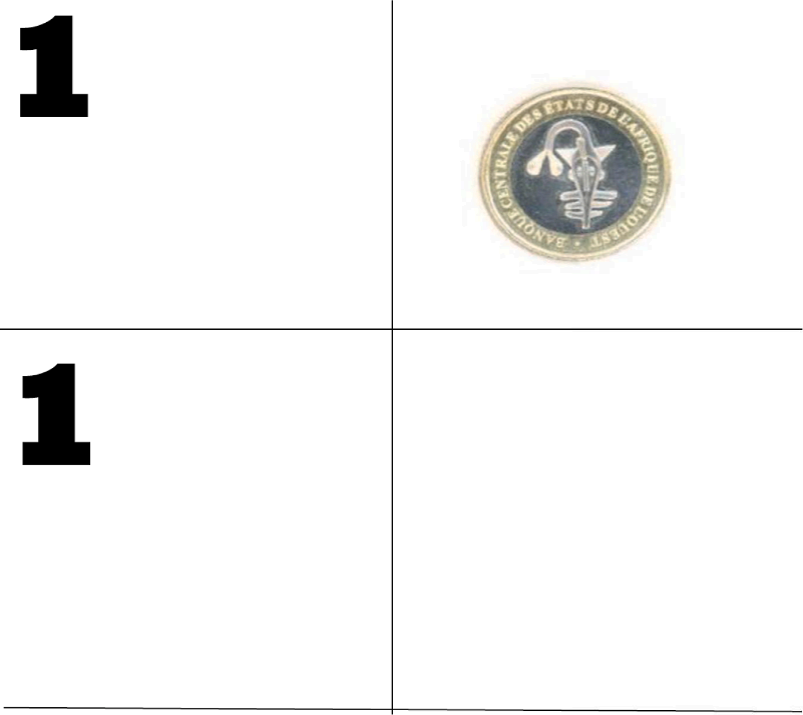
Experiment material: paper token

Conducting laboratory experiments in the field in 121 villages influenced the organization of fieldwork and the design of the experiment itself. Experimenters worked in teams of five, each individual with specific tasks to perform (see Appendix A.2). Given the number of experimental sessions, instructions to the experimenters were made as simple as possible. The experimenters were trained on the protocol for 8 days.^7^ The second challenge, illiteracy, was partly addressed by using a discretized version of the standard public good game. We distributed two paper tokens to participants in each round (see Appendix Fig. A.1): they had to privately choose to put down in the *foroba* either the piece of paper with the *niyoro* picture (*i.e.*, to contribute) or the one without. We explained the base game extensively to make sure that players understood it. At the beginning of each session, the experimenter read the instructions and answered questions until they were sure that all participants fully understood. On average, each experimental session lasted an hour.

The games were incentivized: players earned points that were then translated into rewards. In order not to interfere with the sanitation intervention that took place in some of these villages, we converted the points into small valuable household items unrelated to sanitation (*e.g.* batteries, pens, paper pads, lighters).

### 3.4 Bringing the hypotheses to the data

Based on the game payoffs alone, everyone in the group discussions should be expected to want to convince others to contribute, so that communication is costless (cheap talk) and should not influence contributions. In our context, since community members interact in long-term relationships, contributions in the absence of communication are expected to be high, and promises made during communication sessions may be expected to result in higher contributions.

Throughout the rest of this section, we re-examine the hypotheses from section 2.2 in light of the available data. In particular, we outline the empirical strategy we plan to employ for testing them. To test Hypothesis 1, we use a within-subject design and estimate the before-after difference in contribution rates from Round 1 (no communication) to Round 3 (after participants are exposed to the two communication conditions). The estimate obtained can be interpreted as the effect of communication net of learning effects. As learning effects tend to decrease contributions, the estimate is expected to be an underestimate of the true effect of communication. If this estimate is statistically significant, we are confident that we can reject Hypothesis 1.

For Hypothesis 2, we test whether the learning effects are the same across the two sequences of exposure to communication (directed discussion first followed by undirected discussions, or the opposite). This can be done because we randomly assigned participants to each sequence of communication treatments. We can thus test whether the sequence affected the overall gains obtained in the last round by using comparisons within and between subjects. Not rejecting this hypothesis allows us to design a valid empirical strategy for assessing the relative effectiveness of open versus direct discussions in increasing contribution (Hypothesis 3). In that case, we plan to exploit the between-subject design resulting from the random assignment of participants to one or the other sequence of exposure to the communication conditions to test Hypothesis 3.

For each communication strategy, we observe participants making contribution decisions after being exposed to only one communication condition (in Round 2) and after being exposed to two communication conditions (in Round 3). We can thus assess whether directed (respectively, undirected) discussions are as effective as the combination. To test Hypothesis 4, we take advantage of the between- and within-subject design. If rejected, the participatory programme could be just as effective if it were to implement only one of them.

Lastly, as we randomly selected the community member leading the directed discussions, we can contrast the gains obtained from two opposite types of leaders: randomly selected “champions” who contributed to the public good prior to any instruction and group discussion (in the no-communication condition at Round 1) and randomly-selected “champions” who decided to not contribute in this base round (Hypothesis 5). Considering that the random selection is conducted by village strata, an internal validity issue might arise if leaders who contributed are from villages with higher average contribution levels than those of non-contributing leaders. Hence, we use a difference-in-difference framework to compare gains from no-communication to directed discussion conditions across leader types to test Hypothesis 5. As previously noted, CLTS-trained staff may observe other characteristics such as charisma and the gains from directed contributions may vary also vary with these other characteristics of the leaders, a point to which we return to in the discussion section.

Caution should be exercised regarding the external validity of some of the findings if some members of the community have a strong preference for the practice of OD or do not see value in safe sanitation. In such a setting, selecting a community member at random may result in a poor choice of leader for CLTS. The initial open discussion would be justified as a necessary step for selecting an effective leader and the gains from directed discussions could potentially be larger.

## 4 Empirical findings and discussion

### 4.1 Gains from communication

**Fig. 2:**
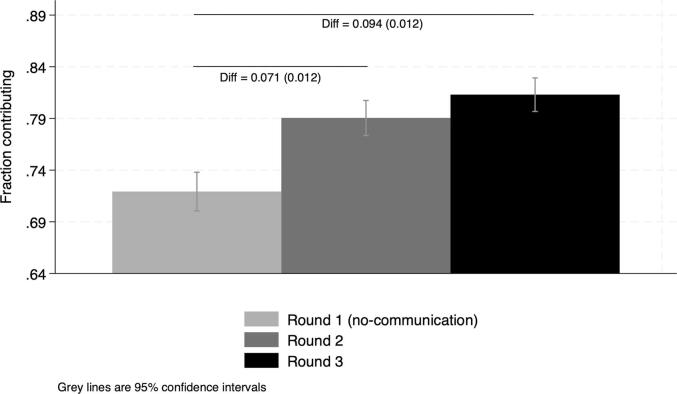
Average contributions across rounds. Note: Reports difference in contribution rates across rounds and robust SE in parenthesis. Round 1: no-communication condition; Rounds 2 and 3: successive communication conditions (**directed** and **undirected** in random order).

**Fig. 3:**
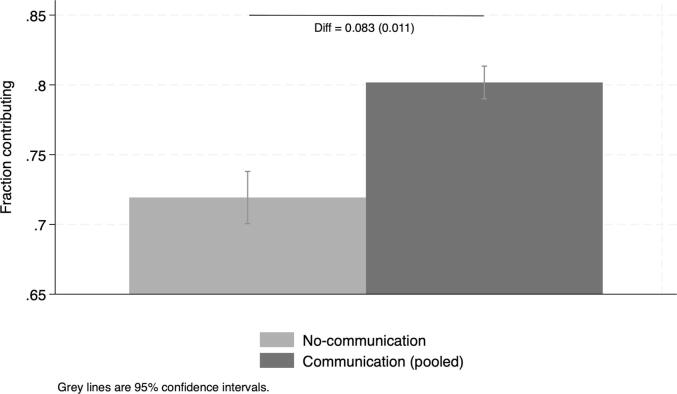
Average contributions: pooled communication conditions vs. no communication. Note: Reports difference in average contribution rates in communication vs. no communication conditions and robust SE in parenthesis.

We observe increases in the contribution rate over time from a level of 72 % in the first round without communication (Fig. 2 and Appendix Table A4. This is a relatively high rate of contributors compared to that of laboratory experiments with university students, but similar to the ones obtained from laboratory experiments in the field in developing countries (Cardenas and Carpenter, 2008).^8^ We find large gains from communication. The average contribution rate is 7.1 percentage points higher in Round 2 and 9.4 percentage points higher in Round 3 compared to Round 1 (Fig. 2 and Appendix Table A4). We then estimate the difference in the average contribution rate between the pooled communication conditions and the no-communication one (Table 5). This within-subject (before-after) comparison provides an estimate of the effect of communication on contributions under the assumption that no other changes affected contributions between rounds of the game. However, a robust finding of the literature is that learning effects typically result in a decrease in contributions in repeated games. We find that the contribution rates are higher under the pooled communication treatment by a significant 8.3 percentage points, a 11.5 percent increase (Fig. 3). Due to learning effects, we expect this value to be an underestimate of the true effect of communication.

**Table 5:** Gains from communication: OLS estimation.

	(1)
Communication	0.0825^∗∗∗^ (0.0113)
Constant	0.719^∗∗∗^ (0.00954)
N	657

Dependent variable takes value 1 if the participant contributes, 0 otherwise. Communication takes value 1 for observations under any communication condition (directed and undirected) pooled across Round 2 and Round 3 and value 0 for observations under the no-communication condition (Round 1). Notes: ***,** and * indicate statistical significance at the 1, 5 and 10 percent level. Standard errors clustered at the village level in parenthesis.

### 4.2 Testing for learning effects through the sequence of exposure to communication conditions

**Fig. 4:**
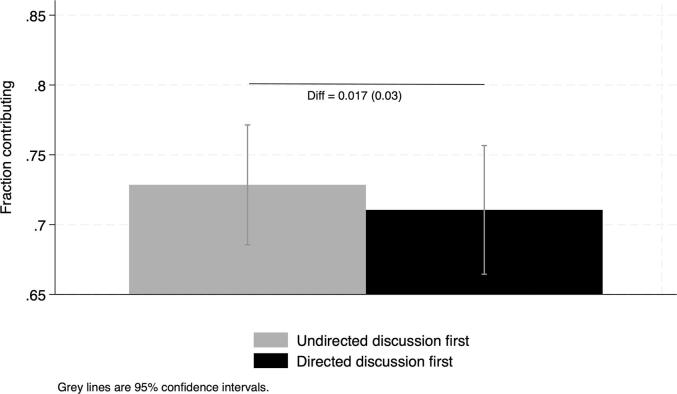
Testing balance across order assignment at Round 1. Note: Reports difference in contribution rates across order assignment at Round 1 (no– communication) with village-level clustered SE in parenthesis. Random order takes value 0 if the community is first exposed to undirected discussions (at Round 2) then directed ones (Round 3) and order takes value 0 for the opposite sequence.

In Fig. 2, the increase in cooperation level is greater between Round 1 and Round 2 than between Round 2 and Round 3 (see also Appendix A4). We observe diminishing marginal returns, consistent with high levels of contributions in the initial game session. These lower gains may also be explained by the fact that as participants become more experienced, the gains tend to decrease. But are learning effects the same across the sequence of exposure to the communication conditions? Lower marginal gains in cooperation may depend on the sequence of treatments through which participants were exposed, *i.e.*, directed followed by undirected group discussion or the opposite. Since we randomized the order of exposure across communities, we can test whether the learning effects are different according to the order assignment. We first check that order assignment is indeed random based on the data collected in Round 1 (no-communication or base condition). We cannot reject that it is not (Table 6 and Fig. 4). The difference in the average contribution rates is small and nonsignificant.

**Table 6:** Checking balance at Round 1.

		(1)
Order		−0.0179
		(0.0318)
Constant		0.728^∗∗∗^ (0.0217)
N		2219

Reports difference in contribution rates across rounds. Round 1: no-communication condition;

Rounds 2 and 3: successive communication conditions (**directed** and **undirected** in random order).

Notes: ***,** and * indicate statistical significance at the 1, 5 and 10 percent level. Standard errors clustered at the village level in parenthesis.

**Fig. 5:**
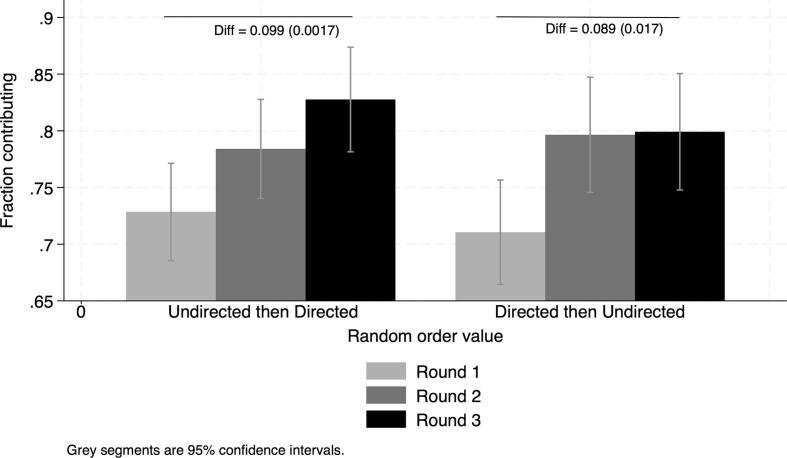
Average contributions across rounds according to the sequence of exposure to communication conditions. Note: Random order takes value 0 if exposed to undirected discussions first and value 1 if exposed to directed discussions first. Reports overall differences in contribution rates and robust SE in parenthesis. Round 1: no-communication condition; Rounds 2 and 3: successive communication conditions (**directed** and **undirected** in random order).

We combine between- and within-subject differences and find no significant differences in gains from Round 1 to Round 3 according to the sequence of exposure to the two communication conditions (Appendix Table A4 and Fig. 5). When exposed to undirected discussions followed by directed ones, the increase in the average contribution rate is a significant 10 percentage points. It is a significant 8.9 percentage point increase when exposed to directed discussions followed by undirected ones. The difference between these gains is not significantly different from zero, indicating that learning effects, if present, are the same across the two sequences of exposure to communication treatments.

### 4.3 Are directed and undirected discussions as effective?

**Fig. 6:**
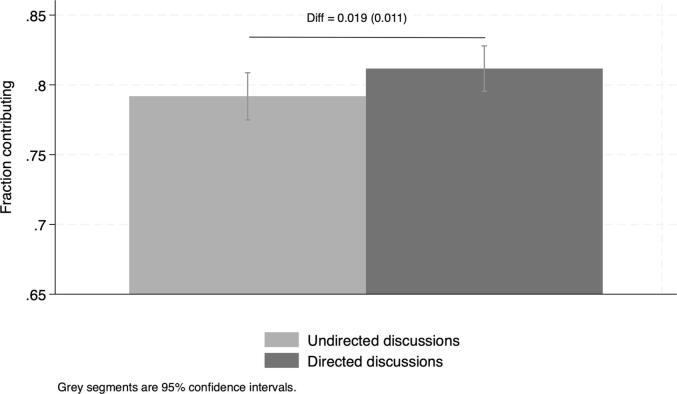
Average contributions across communication conditions. Note: Reports difference in contribution rates across communication conditions and robust SE. Data from Round 2 and Round 3 only (communication conditions). **Undirected discussion** pools Round 2 observations from villages exposed to undirected discussions first and Round 3 observations from villages exposed to directed discussions first. **Directed discussion** pools Round 2 observations from villages exposed to directed discussions first and Round 3 observations from villages exposed to undirected discussions first.

Since learning effects, if present, are the same on average across order assignment, we can compare gains in contributions in undirected vs*.* directed communication conditions.^9^ We restrict the sample to the communication rounds. To construct the set of observations exposed to undirected discussions, we pooled Round 2 observations from villages exposed to undirected discussion first and Round 3 observations from villages exposed to directed discussions first. To construct the set of observations exposed to directed discussions, we pooled Round 3 observations from villages exposed to undirected discussion first and Round 2 observations from villages exposed to directed discussions first. We then estimate the difference in the average contribution rates across communication conditions. In Fig. 6 and Appendix Table A5 we find a significant but small difference across communication conditions (1.9 percentage points higher under directed as compared to undirected discussions). In our experiment, making the collective goal salient through the voice of a community member does not lead to a substantially higher outcome than simple undirected group discussions. Note that since this community champion is randomly picked, we are measuring the effect of the information, keeping the leader quality constant. In CLTS, community champions are not randomly selected, and it is possible that they are better at convincing the other community members to act collectively than the random member.

### 4.4 Value-added from combining different communication methods

**Fig. 7:**
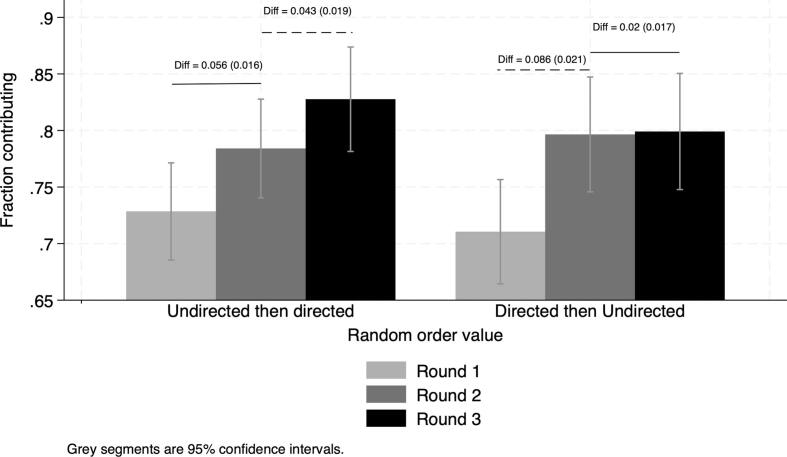
Value-added of combining communication conditions. Note: Random order takes value 0 if exposed to undirected discussions first and value 1 if exposed to directed discussions first. Reports overall differences in contribution rates and robust SE in parenthesis. Round 1: no-communication condition; Rounds 2 and 3: successive communication conditions (**directed** and **undirected** in random order).

To investigate the value-added from combining different communication methods, we exploit the between- and within-subject design. We compare the marginal effects of each communication condition according to the order in which the village is exposed. We make two comparisons. We compare gains from directed discussions when they come first (right- hand side of Fig. 7) to gains when they are preceded by undirected discussions (left-hand side of Fig. 7). Then we compare the gains from the undirected discussions when they come first (left-hand side of Fig. 7) to the gains when they are preceded by the undirected discussions (right-hand side of Fig. 7). The appendix Table A4 reports the corresponding estimates.

We find that directed discussions alone are as effective as the combination of directed and undirected ones. The effect of directed discussions is reduced to a half when they are preceded by undirected ones than when they come first (4.3 vs*.* 8.6 percentage points). In contrast, undirected discussions alone lead to a 5.6 percentage points gain, but there are no gains from undirected discussions when they are preceded by directed ones (0.2 percentage point increase, not statistically significant). Hence, undirected discussions alone are not as effective as a combination of directed and undirected ones.

### 4.5 Community champion in directed discussions

The design of our games was intended to reproduce some relevant aspects of CLTS. Although the community intervention has directed and undirected group discussions as portrayed in the games, there are some important differences that limit the external validity of our findings. In CLTS, the directed discussion was led by a sanitation champion identified by CLTS facilitators while the game leader is chosen randomly in the group of participants. Actual community champions can be expected to have already identified the socially desirable outcome and to be more likely to adhere to it than community members who are not selected by the programme implementors. We test if random leaders who contributed in the base game (under no-communication) led to higher gains in contribution rate in the directed group discussion.

**Fig. 8:**
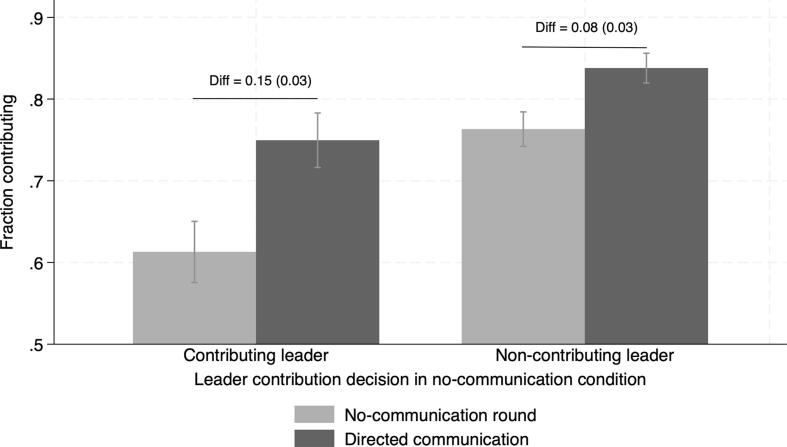
Directed discussion: the effect of a contributing leader. Note: Reports differences in contribution rates in the leader round and no-communication round according to whether leaders contributed or not in the no-communication round and robust SE in parenthesis. Grey segments are 95% confidence intervals.

The findings are reported in Table 7. Column 1 (respectively, column 2) provides a naive estimate of the difference in contribution rates under directed discussions (respectively, no– communication) according to leader type. The former is shown on the right-hand side of Fig. 8 and the latter on the left-hand side of the same figure. Both estimates overstate the effect of a contributing leader in directed discussions (statistically significant and positive differences, respectively of 8.9 and 15.3 percentage points increases). The actual effect is negative, though only statistically significant at the 10 percent level.

**Table 7:** Directed discussion: the effect of a contributing leader.

	(1)	(2)	(3)
Contributing leader	0.0899^∗∗^ (0.0387)	0.153^∗∗∗^ (0.0329)	0.153^∗∗∗^ (0.0329)
Directed discussions			0.137^∗∗∗^ (0.0324)
Directed x Contributing leader			−0.0627^∗^ (0.0367)
Constant	0.748^∗∗∗^ (0.0333)	0.611^∗∗∗^ (0.0283)	0.611^∗∗∗^ (0.0283)
N	2219	2219	4438

Dependent variable takes value 1 if the participant contributes, 0 otherwise. Model (1): restricted to observations under directed discussions;

Model (2): restricted to observations under no-communication;

Model (3): difference-in-difference estimate across type of leader and communication condition. Notes: ***,** and * indicate statistical significance at the 1, 5 and 10 percent level.

Standard errors clustered at the village level in parenthesis.

Given the potential gap between the incentives of a randomly-selected facilitator in the games and in the real-life setting of CLTS, our findings do not allow us to conclude that CLTS should randomly select community members to act as local facilitators. Instead, we interpret our finding as evidence that the intrinsic willingness to contribute is not a good proxy for leader quality.

## 5 Summary and discussion

Community members were found to be 8.3 percentage points more likely to contribute to a local public good under communication, a significant 11.5 percent increase from a proportion of contributors under the no-communication condition of 72 percent. The evidence is in line with existing evidence that contributions increase with communication (see, *e.g.*, Isaac and Walker, 1988, Sally, 1995, Cason and Khan, 1999, Bochet and Putterman, 2009).

Based on within-subject comparisons, contributions were found to increase by 7.1 percentage points from the no-communication round to the first communication round and by 9.4 percentage points from the no-communication round to the last communication round. Since we randomized the order in which subjects were exposed to the two communication conditions (directed first followed by undirected or the opposite), we tested for order effects. We find no evidence that the sequence of communication strategies affected the overall gains obtained in the last round. With no evidence of order effects, we pool similar treatment conditions across rounds to estimate the relative gains from open discussion as compared to directed ones using a between-subject comparison. We find that the gains from directed communication are only slightly higher than the gains from undirected communication (2 percentage points difference).

As CLTS community mobilization is designed as a two-stage process of discussion, we also assess the complementarities between directed and undirected discussions using within-subject comparisons. We find no value added from open discussion for community members who are first exposed to the directed communication strategy and then exposed to undirected discussions. In contrast, there are additional gains for those first exposed to the open group discussions and then exposed to the directed ones. Can a community mobilization achieve the same outcome by relying only on the directed communication strategy facilitated by a randomly-selected community member?

One rationale for the CLTS decision to conduct open discussions before directed ones is to provide space for a community member to present itself as a sanitation champion. We exploited the fact that the community member who directed the discussions in our game setting was randomly selected to test whether the gains from directed communication were greater for facilitators who contributed in the base game before any communication. Leaders who contributed in the absence of communication were not more effective in increasing the contributions of others than those who did not. The effectiveness of directed discussions structured around a single and clear message is also consistent with the asymmetry in the marginal gains to open and directed discussions that we found. Importantly, there is no evidence that selecting a community champion willing to contribute to the local public good is the key to generating the effects. Given that directed discussions alone are as effective as the combination of directed and undirected ones, an effective participatory strategy that values community members’ time should favor the directed communication strategy facilitated by a community member to a two-stage communication strategy. This interpretation should be approached with caution in the absence of agreement regarding the beneficial nature of the local public good.

Additional studies could further explore the heterogeneity in impacts to help identify the characteristics of the most effective influencers. Randomly-selected community members can be expected to be heterogeneous in their susceptibility to be influenced by the prescription to cooperate as well as in their capacity to influence others. Thus, it can be expected that their impact will vary along these dimensions. We limited the exploration to one dimension because of the small number of ‘champions’ (1 per community). In Hypothesis 5, we ask if gains from directed communication vary according to whether the randomly-selected leader contributed in the initial round in the absence of any communication. This focus allows us to question the CLTS approach which emphasizes the role of initial open discussions on sanitation as a necessary step in their participatory process to provide space for a community member to spontaneously emerge as a natural leader.

With a large number of “champions” (*e.g.*, multiple randomly selected leaders per community), one could identify the effect of a contributing leader using a model with village fixed effects, controlling for the intra-village correlation in characteristics and behavior of participants. In addition, one could also test if the propensity to contribute of the randomly selected “champions” is higher when instructed to act as facilitators compared to the one in the absence of communication. Even if on average they are more likely to contribute when designated as facilitators, their responses to the prescription to contribute may be heterogeneous according to their own social preferences, as well as to perceived social benefit and cost of giving their support to the prescription. Secondly, one could check if the impact of facilitation depends on the champions’ ability to influence others, which can be expected to be correlated with their position in the village. Individuals in a position of authority, more central individuals can be expected to yield higher impacts conditional on their level of responsiveness to the prescription to contribute. In a design with a sufficiently large number of randomly selected facilitators, the heterogeneity in impact can be explored with machine learning techniques to select the characteristics that best predict the heterogeneity in their effectiveness (Belloni et al., 2014b, Belloni et al., 2014a).

## 6 Conclusions

In this paper, we report experimental findings from a series of public good games played in the field under conditions designed to mimic the operations of a community-based sanitation intervention. Our paper thus contributes to a large literature discussing the benefits and limitations of participatory approaches (Casey, 2018).

We examined the workings of participation in the widely praised CLTS sanitation intervention. Community mobilization is the key activity in this intervention. We identified two distinct processes in the community mobilization for the Mali CLTS: (1) the triggering process facilitated by a programme implementor who introduces the subject (sanitation) through various activities and allows an undirected public discussion to take place; (2) delegation by the external facilitator of her authority to direct the group discussions to a community champion.

Our experimental findings support the claim that community discussions improve cooperation in the collective action problem setting, although we find little difference between the two types of communication strategies. We find that directed discussions alone are as effective as the combination of directed and undirected ones, while undirected discussions alone are not. This suggests that CLTS may be just as effective through a directed discussion session. When there is broad agreement on the importance of improved sanitation, the process of identifying a community champion that could have justified the first stage of undirected public discussions may not be necessary; in this case, making the collective goal and individual actions to achieve it salient is the driving factor underlying the gains from directed discussions.

## CRediT authorship contribution statement

**Maria Laura Alzua:** Methodology, Investigation, Funding acquisition. **Juan Camilo Cardenas:** Methodology. **Habiba Djebbari:** Writing – review & editing, Writing – original draft.

## Declaration of competing interest

The authors declare that they have no known competing financial interests or personal relationships that could have appeared to influence the work reported in this paper.

## Figures and Tables

**Table A1:** Timeline of activities Date.

Activities	
April-June 2011	Baseline experimental games and survey
September 2011-June 2012	Implementation of the CLTS intervention
April-June 2013	Endline experimental games and survey

**Table A2:** Composition of the experimenter team.

Team member	Role
Recruiter	Keeping track of the identity of the participants Explaining the games
Facilitator Accountant Observer Supervisor	Determining and distributing material rewards Checking that participants follow protocol Supervising the team of experimenter

**Table A3:** Descriptives statistics on game participants.

	Mean
	(SD)
Number of game participants	18.49
	(3.80)
Speak bambara	0.73
	(0.41)
Head of household	0.24
	(0.15)
Spouse of head	0.69
	(0.16)
Other household member	0.07
	(0.09)
Male	0.25
	(0.16)
Muslim	0.86
	(0.20)
Married	0.93
	(0.07)
Occupation: Farming	0.24
	(0.21)
Occupation: herding	0.02
	(0.05)
Occupation: merchant	0.03
	(0.06)
Occupation: construction	0.07
	(0.10)
Occupation: forestry	0.06
	(0.09)
Wage labor	0.01
	(0.03)
Family labor	0.62
	(0.20)
Self-employed	0.37
	(0.20)
Usually work but not in the last week	0.27
	(0.36)
Weekly number of hours of work	36.77
	(8.70)
Locus of control index	−0.28
	(0.21)
Social capital index	2.89
	(0.30)
No. of organisation	2.09
	(0.59)
No. of position of power	0.77
	(0.38)
Household asset index	0.44
	(0.07)
Farm asset index	0.57
	(0.10)
Livestock index	21.73
	(11.34)
No. of observations	121

**Table A4:** Average contributions across rounds and order.

	Round 1	Round 2	Round 3	Test	Difference	t-statistic
Order = 0						
Mean	0.728	0.784	0.827	*H*_0_: *Y*^2^ = *Y*^3^	0.043**	t = 2.53
SD	0.444	0.411	0.377	*H*_0_: *Y*^1^ = *Y*^2^	0.056***	t = 3.04
No. obs.	1079	1079	1079	*H*_0_: *Y*^1^ = *Y*^3^	0.099***	t = 5.58
Order = 1						
Mean	0.710	0.796	0.799	*H*_0_: *Y*^2^ = *Y*^3^	0.003	t = 0.17
SD	0.453	0.402	0.400	*H*_0_: *Y*^1^ = *Y*^2^	0.086***	t = 4.79
No. obs.	1140	1140	1140	*H*_0_: *Y*^1^ = *Y*^3^	0.089***	t = 4.97
All						
Mean	0.719	0.790	0.812	*H*0: *Y_._*^2^ = *Y_._*^3^	0.02*	t = 1.83
SD	0.449	0.407	0.390	*H*0: *Y_._*^1^ = *Y_._*^2^	0.07***	t = 5.51
No. obs.	2219	2219	2219	*H*0: *Y_._*^1^ = *Y_._*^3^	0.09***	t = 2.95

Notes: Order = 0 is **undirected discussions** followed by **directed discussions**; Order = 1 is the opposite. *Y ^i^* is the average contribution at round *i* for order *j*. *Y_._^i^* is the average contribution at round *i*. Note: ***,** and * indicate statistical significance at the 1, 5 and 10 percent level.

**Table A5:** Average contributions under directed and undirected discussions.

	Average Contribution	SD	No. Obs.
Directed discussions	0.811	0.391	2219
Round 2, Order = 1	0.796	0.402	1140
Round 3, Order = 0	0.827	0.377	1079
Undirected discussions	0.791	0.406	2219
Round 2, Order = 0	0.784	0.411	1079
Round 3, Order = 1	0.799	0.400	1140
Test Difference	*H*_0_: Directed = Undirected 0.02*	0.011	4438
Statistic	t = 1.67		

## Data Availability

Data will be made available on request.

## Appendix

.

Notes: Order = 0 is **undirected discussions** followed by **directed discussions**; Order = 1 is the opposite. Note: * indicates statistical significance at the 10 percent level.

## Footnotes

1 In previous work on CLTS Mali, we found substantial improvements in child growth outcomes (Pickering et al., 2015) and sanitation behavior (Alzúa et al., 2020).

2 In this community-based program, there are no funds or in-kind benefits to allocate across projects or households, and the role of external facilitators, employed by CLTS, is minimal.

3 In previous work, Alzúa et al. (2020) found a large and significant increase in social disapproval against open defecation as a result of CLTS.

4 CLTS was first implemented in Bangladesh in 1999 and is now present in more than 50 countries in the global South. At least 20 countries adopted CLTS as their national sanitation strategy for rural areas. CLTS has wide support from international organizations (*e.g.* World Bank, UNICEF, WaterAid) as an effective participatory solution to improve sanitation.

5 In the smallest communities, field enumerators were asked to recruit more than 50% of eligible households.

6 Transcript is as follows: “*The group of a whole gets the highest number of points if everyone contributes. The group gets the lowest number of points if no one does. You can now try to convince the other participants to contribute so that the group gets the highest number of points.*”.

7 Full protocol available upon request.

8 In Zimbabwe, Barr (2001) find that participants to a VCM contribute between 48 and 52% of endowment. In Vietnam and Thailand, it is between 72 and 76% and between 61 and 73% (Carpenter et al. 2004). In Kenya, Ensminger (2000) found that people contribute 58% of their endowment. In Peru, Karlan (2005) found that 81% of participants contribute in a threshold public game. Figures for the US are lower (between 30–40%).

9 This comparison is not against the base condition without communication. It only allows estimating the gains from one communication condition against the other.
